# Healthcare mission to a humanitarian disaster: a descriptive study of multidisciplinary interventions during the 2024 floods in Rio Grande do Sul, Brazil

**DOI:** 10.31744/einstein_journal/2026AO2192

**Published:** 2026-09-02

**Authors:** Tarso Augusto Duenhas Accorsi, Renata Albaladejo Morbeck, Claudia Candido da Luz, Fabio de Castro Jorge Racy, Flavio Tocci Moreira, Karen Francine Köhler, Carlos Henrique Sartorato Pedrotti

**Affiliations:** 1 Hospital Israelita Albert Einstein Telemedicine Department São Paulo SP Brazil Telemedicine Department, Hospital Israelita Albert Einstein, São Paulo, SP, Brazil.; 2 Hospital Israelita Albert Einstein Medical Assistance Group for Disasters and Catastrophes São Paulo SP Brazil Medical Assistance Group for Disasters and Catastrophes, Hospital Israelita Albert Einstein, São Paulo, SP, Brazil.

**Keywords:** Flood disaster, Humanitarian mission, Multidisciplinary care, Emergency response, Primary healthcare

## Abstract

**Objective:**

Floods are the most common natural disasters worldwide, and their health impacts are exacerbated by climate change and population vulnerability. Humanitarian missions are essential for providing rapid and sustained medical care in disaster settings. However, evidence regarding the clinical and procedural aspects of multidisciplinary care during such missions remains limited. We examined the clinical profile and interventions provided during a humanitarian mission conducted in response to severe flooding in southern Brazil.

**Methods:**

This retrospective observational study was conducted during a humanitarian mission organized by *Hospital Israelita Albert Einstein* in Canoas, one of the municipalities most severely affected by the 2024 floods in Rio Grande do Sul. From May to June 2024, all individuals who voluntarily sought medical care were included. Multidisciplinary teams provided in-person assistance. Data from manual medical records were analyzed using descriptive statistics.

**Results:**

A total of 2,060 patients were evaluated. General practitioners conducted 33.16% of consultations. The most frequent procedures were dermatological, including wound care (72.69%) and abscess drainage (7.63%). Point-of-care testing primarily targeted dengue (50.58%) and influenza (19.77%), with 54.15% of tests yielding positive results. The most frequently administered medications were analgesics (34.61%), bronchodilators (8.98%), and antibiotics (8.14%). Mental health consultations accounted for 6.46% of total visits. Most patients (94.34%) were discharged, whereas 3.28% required referral to emergency care.

**Conclusion:**

The mission demonstrated the effectiveness of a rapid multidisciplinary care model in addressing acute healthcare needs during flooding. Integrating diagnostic testing and mental healthcare may help reduce disease burden and alleviate pressure on local healthcare systems.

## Highlights

Most acute flood-related presentations were managed on site.

Wound care predominated among post-flood procedures.

Point-of-care testing prioritized dengue and influenza.

Psychological support was integrated into frontline disaster care.

## In Brief

A retrospective descriptive analysis of a multidisciplinary floodresponse mission in southern Brazil found that over 2,000 on-site assessments addressed mainly low- to moderate-complexity conditions, with frequent wound care, dengue/influenza testing, psychological support, and high discharge after evaluation, supporting local surge capacity.

## Graphical abstract

**Figure:**
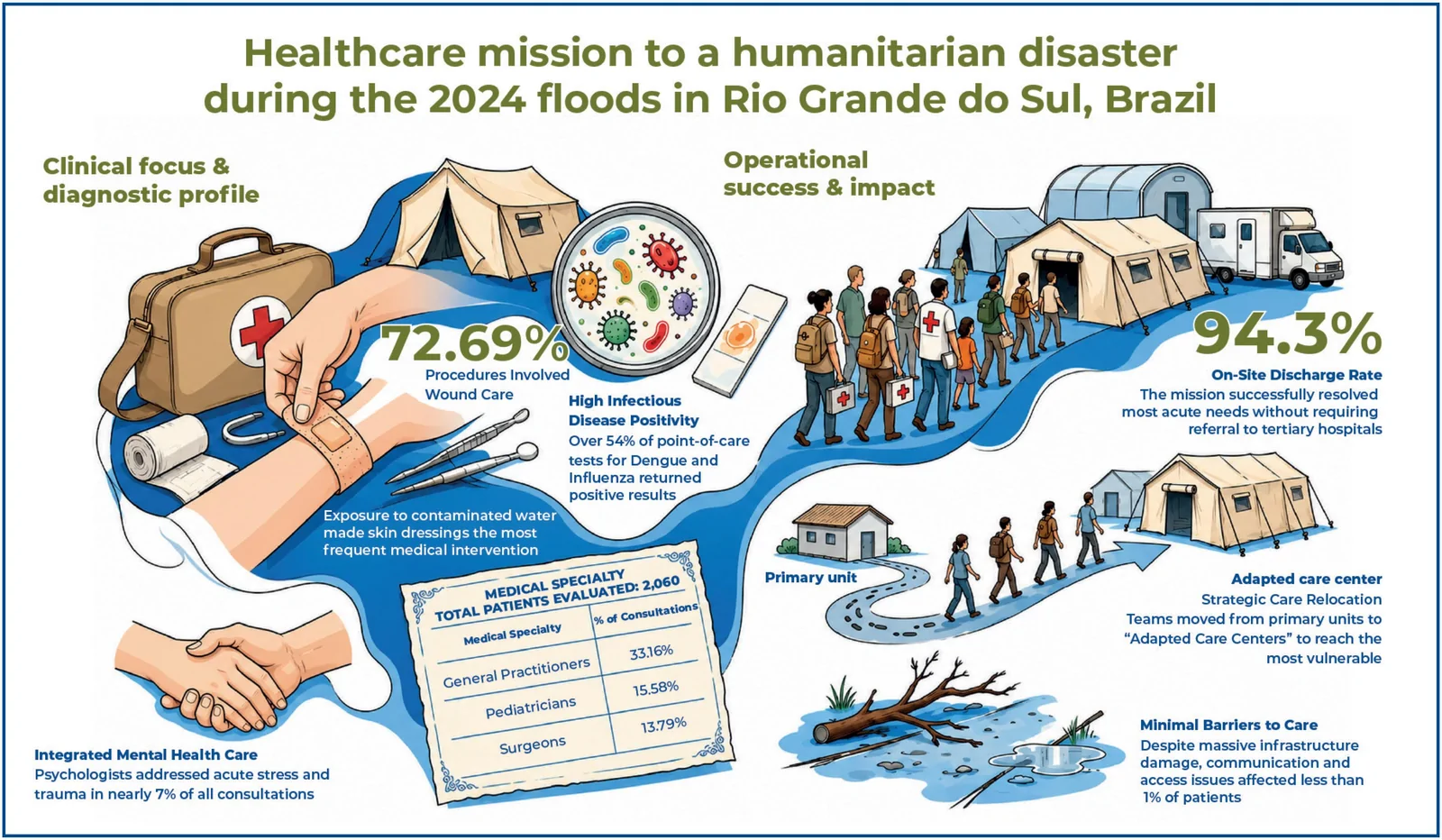

## INTRODUCTION

Globally, floods are the most frequent natural disasters, and their impacts are expected to increase because of climate change and population dynamics. Flooding poses immediate risks to human health and long-term consequences associated with displacement and deteriorating living conditions.^(1)^ The health burden of flood-related disasters is extensive and includes skin and soft tissue infections, gastroenteritis, and zoonotic diseases such as leptospirosis, while also exacerbating noncommunicable diseases and disrupting healthcare infrastructure.^(2)^

Humanitarian missions play an important role in mitigating the effects of flood-related emergencies by addressing both immediate and long-term health challenges.^(2)^ Previous studies indicate that medical teams are essential for the early recognition and management of shock and infection in severely affected individuals, which are fundamental components of post-disaster medical care.^(3)^ In addition, these teams contribute to the prevention, diagnosis, and treatment of hyperthermia, vector-borne diseases, tropical dermatologic conditions, infectious diarrhea, and injuries caused by animals or insects.^(4)^

Beyond acute care, humanitarian health interventions provide important short- and long-term benefits, including reduced mortality, prevention of disease outbreaks, alleviation of suffering, and strengthening of local healthcare systems.^(5)^ The effectiveness of these missions depends on rapid deployment, efficient logistical coordination, adherence to international medical protocols, and targeted investment in cost-effective interventions.^(6)^

However, few studies have specifically evaluated patterns of multidisciplinary healthcare delivery during humanitarian missions in flood settings. The clinical characteristics of treated patients, types of procedures performed, and degree of clinical resolution remain insufficiently characterized.

## OBJECTIVE

In this study, we aimed to analysed the clinical profile and management of patients evaluated during a humanitarian health mission conducted in response to a flood-related disaster.

## METHODS

### Study design and participants

This retrospective descriptive study was conducted at *Hospital Israelita Albert Einstein*, where the humanitarian mission was organized. Healthcare professionals were deployed to a designated healthcare facility and provided multidisciplinary care according to institutional protocols. Study coordination and data analysis were performed at the telemedicine center. All authors approved the manuscript submission and were responsible for ensuring the integrity and accuracy of the data and adherence to predefined study protocols.

Between May and June 2024, anonymized data were collected from patients evaluated during the humanitarian mission in Canoas. Adult patients (≥18 years) who voluntarily sought medical care at the mission healthcare facility were included. Patients without documented medical records were excluded.

This study was approved by the Institutional Review Board of *Hospital Israelita Albert Einstein* (CAAE: 83618324.5.0000.0071; # 7.376.150). Given the retrospective design and use of anonymized data, the requirement for informed consent was waived.

### The humanitarian disaster

Between late April and early May 2024, extreme rainfall caused catastrophic flooding in Rio Grande do Sul. The event was described as the largest climate-related disaster in the state's history and affected approximately 2.4 million people, including 442,000 displaced individuals. The floods resulted in 183 confirmed deaths, 806 injuries, and 27 missing persons. Approximately 478 municipalities sustained urban and rural infrastructure damage. Rainfall reached unprecedented levels, with 500-700mm recorded within 5 days in some regions, corresponding to nearly one-third of the annual average precipitation. In Porto Alegre, 540mm of rain was recorded in May, exceeding the previous historical record of 447mm.^(7)^

The floods caused extensive disruption to transportation, electricity, water supply, and telecommunications systems. More than 400,000 buildings were affected, including 44,600 destroyed homes. Estimated economic losses exceeded R$10 billion, primarily affecting housing, public infrastructure, and agriculture. State and federal governments declared a public calamity and allocated emergency resources for rescue and reconstruction efforts. The federal government released R$600 million in emergency aid and R$51 billion in subsidized reconstruction credit. The Brazilian Armed Forces deployed 30,000 personnel, 17 aircraft, 84 boats, and 385 vehicles to support rescue operations. International assistance was also provided by multiple countries.^(8)^

### Humanitarian mission of *Hospital Israelita Albert Einstein*

The hospital team included physicians, nurses, psychologists, social workers, respiratory therapists, and logistics specialists. The mission aimed to restore medical care in the areas of Canoas most severely affected by the floods. According to the Brazilian Institute of Geography and Statistics, the municipality had an estimated population of 347,657 in 2022 and is located 19km from Porto Alegre, with a territorial area of 130.79km².

The multidisciplinary team from *Hospital Israelita Albert Einstein* arrived in Canoas during the peak emergency response phase. An emergency care unit was established within the Guajuviras Primary Health Unit and remained operational for 3 weeks (Figures 1 and 2). This intervention enabled local Family Health Strategy teams to redirect efforts toward restoration of routine primary healthcare services.

**Figure 1:**
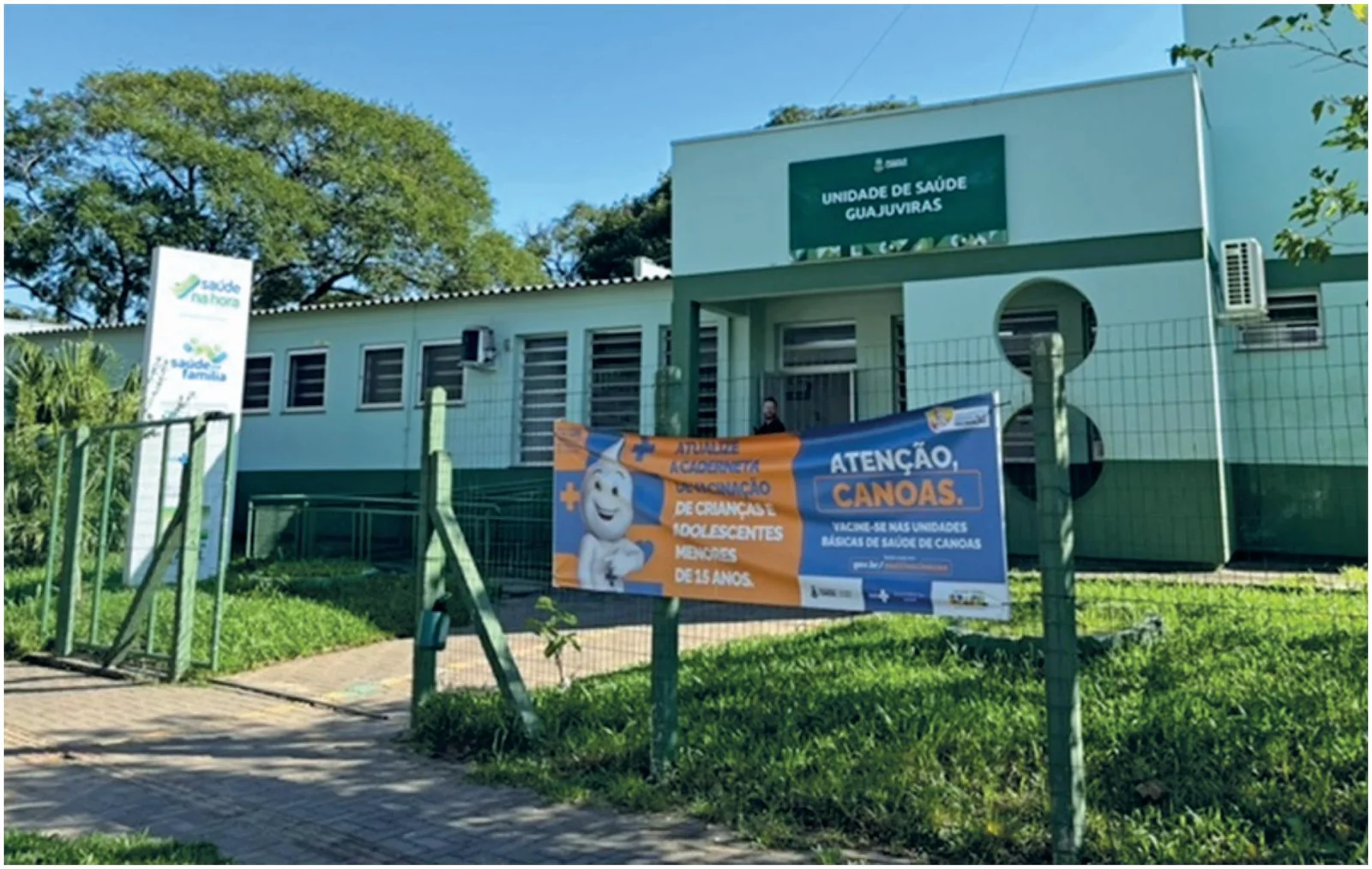
Location of the first emergency care unit implemented

**Figure 2:**
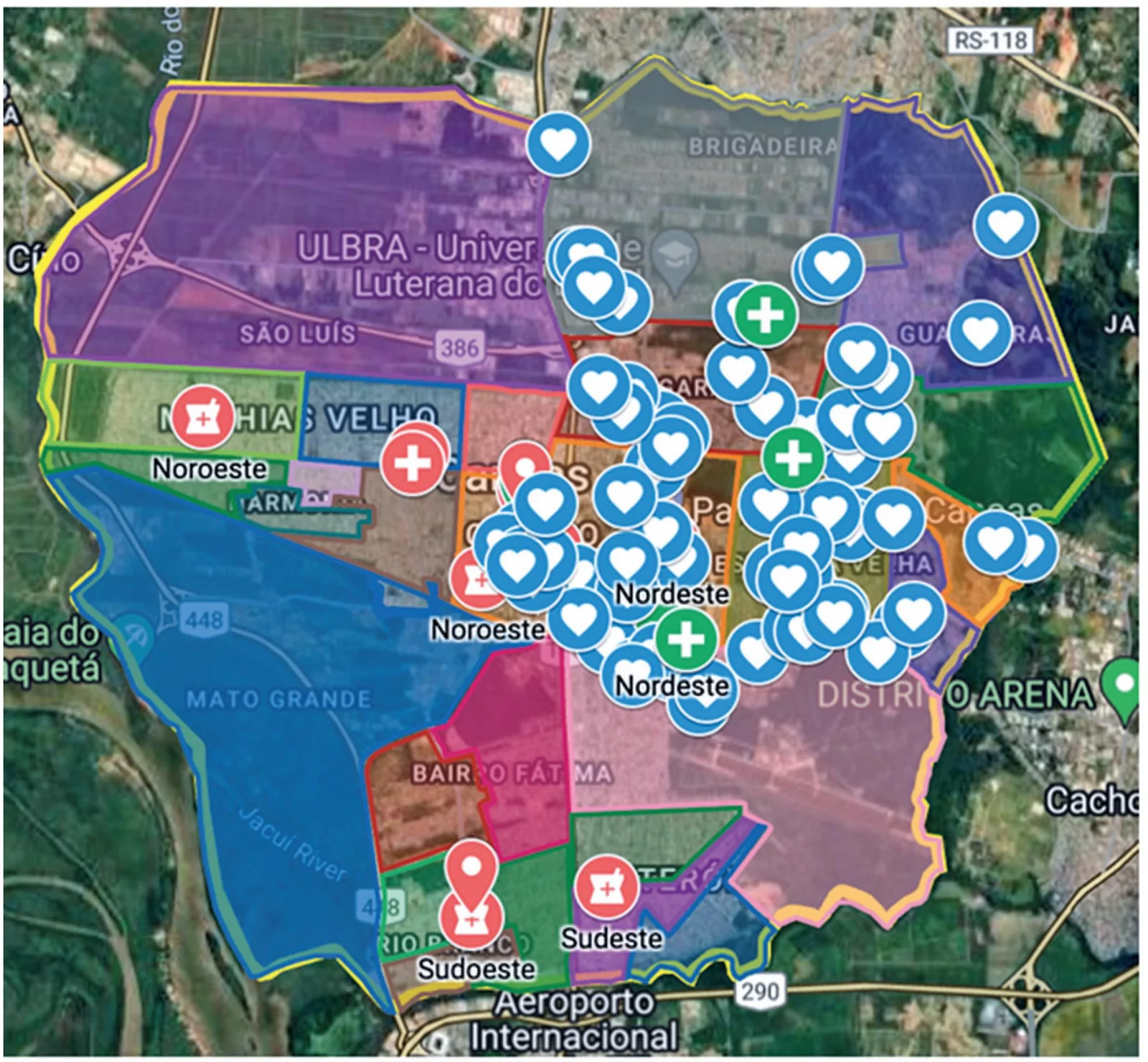
Location of mission facilities on the local map

Following partial restoration of the original health facility, an adapted care center was established near the most heavily affected areas. The site was strategically selected because of the greater clinical complexity of cases encountered and the inoperability of nearby healthcare facilities caused by flooding (Figures 2 and 3). This initiative ensured access to essential medical care for a highly vulnerable and underserved population.

**Figure 3:**
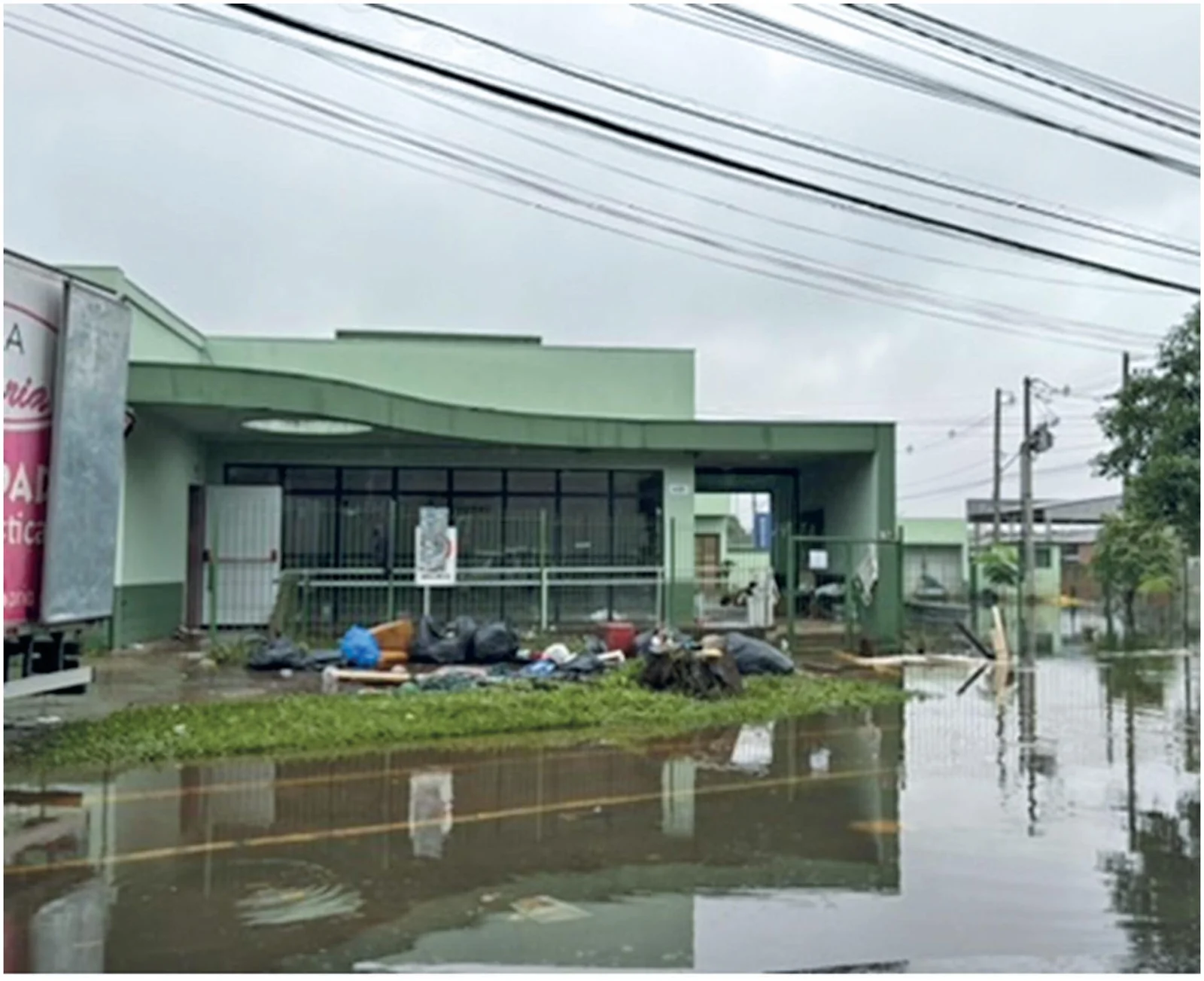
Location of adapted care center

The main activities performed during the mission included: (a) Triage and emergency care, including patient assessment and prioritization of severe cases; (b) Disease prevention measures aimed at reducing outbreaks of flood-related conditions such as leptospirosis, dengue, gastrointestinal infections, and respiratory infections.

### Primary outcomes

Primary outcomes included reasons for seeking care, diagnoses established during consultation, treatments prescribed, diagnostic tests requested, and demographic and clinical characteristics of the patients.

### Data extraction

Because of the catastrophic conditions during the disaster response, patient data were recorded manually in medical charts. Mission team members subsequently tabulated and analyzed the data.

### Statistical analysis

Statistical analyses were performed using IBM SPSS Statistics for Windows, version 22.0 (IBM Corp., Armonk, NY, USA). The analysis was descriptive only. Categorical variables are presented as counts and percentages.

## RESULTS

A total of 2,059 participants were included in the analysis. The mean age was 34.2±19.8 years. Regarding age groups, 1,312 participants (63.7%) were adults (18-59 years), 498 (24.2%) were children or adolescents (<18 years), and 249 (12.1%) were older adults (≥60 years). Females accounted for 1,147 participants (55.7%), whereas 912 (44.3%) were males. At the time of assessment, 782 participants (38.0%) were residing in shelters and 1,277 (62.0%) were not. Among sheltered participants (n=782), 320 (40.9%) were staying at the "Carlos Drummond de Andrade" shelter, 277 (35.4%) were staying with relatives or friends, and 185 (23.7%) were residing in facilities such as churches, schools, or daycare centers (Table 1).

**Table 1:** Participant characteristics

Characteristic		Value
Age, years		
Mean±standard deviation		34.2±19.8
Age category, n (%)		
Children/adolescents (<18 years)		498 (24.2)
Adults (18–59 years)		1,312 (63.7)
Older adults (≥60 years)		249 (12.1)
Sex, n (%)		
	Male	912 (44.3)
	Female	1,147 (55.7)
Residence in shelter, n (%)		
	Yes	782 (38.0)
	No	1,277 (62.0)
Shelter type (among sheltered participants, n=782), n (%)		
	"Carlos Drummond de Andrade" shelter	320 (40.9)
	Church/school/daycare facilities	185 (23.7)
	Relative's/friend's home	277 (35.4)

Healthcare providers assessed 2,060 individuals. General practitioners performed 683 assessments (33.16%), followed by pediatricians with 321 (15.58%), surgeons with 284 (13.79%), social workers with 186 (9.03%), respiratory therapists with 172 (8.35%), orthopedists with 144 (6.99%), nurses with 137 (6.65%), and psychologists with 133 (6.46%). Overall, 602 participants (29.22%) reported direct involvement in disaster-related assistance activities, whereas 661 (32.09%) reported no involvement; status was unknown for 797 participants (38.69%). Barriers to consultation were uncommon, with communication-related and infrastructure-related issues reported in 1 (0.05%) and 3 (0.15%) cases, respectively. Limited healthcare facility resources were reported in 1,967 cases (95.48%), whereas no limitations were reported in 93 cases (4.51%) (Table 2).

**Table 2:** Main characteristics of mission assessments.

Characteristic		n (%)
Healthcare provider assessment, n %		2,060
	General practitioner	683 (33.16)
	Pediatrician	321 (15.58)
	Surgeon	284 (13.79)
	Social worker	186 (9.03)
	Physiotherapy	172 (8.35)
	Orthopedist	144 (6.99)
	Nurse	137 (6.65)
	Psychologist	133 (6.46)
Direct involvement in disaster-related assistance		
	Yes	602 (29.22)
	No	661 (32.09)
	Unknown	797 (38.69)
Barrier to consultation		
	Communication	1 (0.05)
	Infrastructure	3 (0.15)
Reported insufficient facility resources		
	Yes	1,967 (95.48)
	No	93 (4.51)

A total of 172 point-of-care tests were requested. Dengue was the most frequently tested condition, accounting for 87 tests (50.58%), followed by influenza with 34 (19.77%), gastroenterocolitis with 16 (9.30%), syphilis with 2 (1.16%), and other conditions with 33 (19.19%). Overall, 189 tests (54.15%) yielded positive results, whereas 160 (45.85%) were negative.

A total of 228 procedures were performed. Most participants underwent wound dressing (181 [72.69%]), followed by abscess drainage in 19 (7.63%), immobilization in 16 (6.43%), wound debridement in 7 (2.81%), suturing in 3 (1.20%), and other procedures in 23 (9.24%).

Local medication administration was recorded in 519 cases. Analgesics were the most frequently administered medications, accounting for 289 administrations (34.61%), followed by antihistamines with 115 (13.77%), anti-inflammatory agents with 77 (9.22%), bronchodilators with 75 (8.98%), antibiotics with 68 (8.14%), intravenous hydration with 68 (8.14%), corticosteroids with 52 (6.23%), and other medications with 91 (10.90%).

Regarding patient outcomes, 1,351 participants (94.34%) were discharged after evaluation, 34 (2.37%) were referred to specialist care, and 47 (3.28%) were referred to the emergency department. Among those referred to the emergency department, 30 (63.83%) used their own means of transportation, whereas 17 (36.17%) required ambulance transport (Table 3).

**Table 3:** Main outcomes

Characteristic		n (%)
Requested point-of-care tests		n=172
	Dengue	87 (50.58)
	Influenza	34 (19.77)
	Gastroenterocolitis	16 (9.30)
	Syphilis	2 (1.16)
	Others	33 (19.19)
Point-of-care test results		
	Positive	189 (54.15)
	Negative	160 (45.85)
Procedures performed		n=228
	Wound dressing	181 (72.69)
	Wound debridement	7 (2.81)
	Abscess drainage	19 (7.63)
	Immobilization	16 (6.43)
	Suturing	3 (1.20)
	Others	23 (9.24)
Local medication administration		n=519
	Analgesic	289 (34.61)
	Anti-inflammatory	77 (9.22)
	Antibiotic	68 (8.14)
	Antihistamine	115 (13.77)
	Bronchodilator	75 (8.98)
	Corticosteroid	52 (6.23)
	Intravenous hydration	68 (8.14)
	Others	91 (10.90)
Outcome related to clinical, orthopedic, surgical, or pediatric care		n=1,432
	Discharge	1,351 (94.34)
	Specialist referral	34 (2.37)
	Emergency department referral	47 (3.28)
	Own transportation	30 (63.83)
	Ambulance transport	17 (36.17)

## DISCUSSION

During the short-term mission, government response strategies were still being developed; however, many people sought medical care spontaneously. The mission provided multidisciplinary care for a variety of urgent conditions, with most patients receiving treatment and being safely discharged after evaluation, simple procedures, or medication administration.

Timely medical interventions can significantly reduce fatalities caused by trauma, infections, and preventable conditions. Studies indicate that displaced populations, including refugees and internally displaced persons, experience high mortality rates due to treatable conditions such as malnutrition, diarrheal diseases, respiratory infections, measles, and malaria.^(9)^ Effective responses that provide clean water, sanitation, shelter, and essential medical services may help mitigate increases in post-disaster mortality. Historical examples, such as the 1994 cholera outbreak in the Goma refugee camps, which claimed nearly 50,000 lives within a month because of delayed interventions, highlight the importance of prompt action. Modern responses use pre-established protocols and rapid mobilization to avert such catastrophic losses.^(10)^

In addition to immediate lifesaving interventions, humanitarian missions contribute to restoring essential healthcare services, including maternal and child healthcare, chronic disease management, and emergency surgery. Six months after the Haiti earthquake, assessments showed that access to healthcare had improved compared with pre-disaster levels because of international medical aid.^(11)^ Emergency health networks frequently provide free care to underserved populations, ensuring access to medical services that would otherwise be unavailable. Furthermore, prolonged crises require nutritional rehabilitation and reproductive health interventions, such as the Minimum Initial Service Package, to maintain health and well-being during instability. These interventions may help reduce the long-term adverse effects of disasters on population health.

Psychological distress is an unavoidable consequence of humanitarian crises, requiring integrated mental health and psychosocial support. Contemporary humanitarian missions follow the Inter-Agency Standing Committee guidelines, which include psychological first aid, safe spaces for children, community engagement programs, and specialized mental healthcare. For example, psychosocial support for survivors and their families during the Ebola outbreak helped reduce stigma and facilitated social reintegration.^(12)^ Addressing emotional trauma may strengthen community resilience and promote more stable post-crisis recovery. Approximately 8% of the mission's services were delivered to patients who actively sought psychological assistance, highlighting the importance of mental health support in humanitarian responses.

Humanitarian missions were initially centered on emergency relief; however, they may also create lasting benefits by improving local healthcare infrastructure and providing professional training. Local healthcare workers frequently collaborate with international teams, gaining skills in trauma care, disease surveillance, and emergency management. Temporary medical facilities may later become permanent healthcare centers, and public health education initiatives may improve community preparedness for future crises. These long-term investments may contribute to more resilient healthcare systems capable of responding more effectively to future emergencies. The model implemented during this mission may have influenced the surrounding healthcare system and potentially improved preparedness for rapid local responses to future emergencies; however, this impact was not directly measurable.

Operational efficiency in humanitarian health missions depends on well-coordinated logistics, which may account for 60%-80% of response costs. Key logistical components include rapid mobilization and transportation, medical supply and supply-chain management, self-sufficiency of medical teams, and compliance with internationally recognized protocols that ensure quality care, ethical standards, and operational efficiency.^(13)^ The high proportion of patients discharged without referral indicates the mission's ability to address the most urgent medical needs on-site within the disaster setting. In humanitarian environments, rapid assessment and treatment of low- to moderate-complexity conditions are essential to avoid overburdening local hospitals and emergency departments. Therefore, the observed care pattern highlights the operational role of field medical teams in providing prompt, accessible care and triaging patients who required more advanced medical attention.

The findings from this humanitarian mission highlight the important role of multidisciplinary emergency medical teams in responding to large-scale natural disasters. The low referral rate to emergency departments (3.28%) further demonstrates the operational capacity of the deployed healthcare model to manage a wide range of acute conditions on-site. Rather than indicating clinical superiority, this finding suggests that the structure and composition of the multidisciplinary team were sufficient to address most presenting complaints within the temporary care setting. This approach aligns with established disaster-response frameworks, in which frontline providers conduct rapid assessments, perform basic procedures, and deliver initial treatment while identifying the relatively small number of cases requiring escalation to tertiary care.

The high frequency of dermatological procedures, such as wound care (72.69%) and abscess drainage (7.63%), is consistent with post-flood epidemiological patterns, in which exposure to contaminated water increases the risk of soft tissue infections and vector-borne diseases. Similarly, the frequency of point-of-care testing for dengue (50.58%) and influenza (19.77%) reflects the epidemiological need to identify and manage infectious diseases that commonly emerge after flooding events. Given that more than half of the tested individuals (54.15%) had positive results, the mission's rapid diagnostic capability may have been important for guiding targeted therapeutic interventions and reducing disease transmission.

Medication administration data provide further insight into the prevailing clinical needs. The predominance of analgesics (34.61%) and anti-inflammatory agents (9.22%) suggests that musculoskeletal complaints and pain syndromes were common among affected individuals, potentially exacerbated by displacement, physical exertion, and poor living conditions. The use of antibiotics (8.14%) highlights the role of antimicrobial therapy in treating bacterial infections associated with unsanitary environments, whereas the administration of bronchodilators (8.98%) and corticosteroids (6.23%) may reflect exacerbations of pre-existing respiratory diseases, which are recognized consequences of flood-related environmental exposure.

Psychosocial support remains a cornerstone of humanitarian medical interventions, as reflected by the involvement of psychologists in 6.46% of patient encounters. Disasters of this magnitude inevitably result in psychological distress, including acute stress reactions, anxiety, and post-traumatic stress disorder. Integrating mental health services into the mission's scope of practice may represent an important shift towards holistic disaster medicine, ensuring that both physical and psychological health needs are addressed.

The World Health Organization developed the Health Emergency and Disaster Risk Management framework, which emphasizes preparedness, coordinated response, and health-system resilience throughout the entire disaster cycle, including mitigation, response, and recovery phases.^(14)^ Within this framework, emergency medical teams play a central role in delivering rapid and organized medical assistance during large-scale emergencies, supporting overwhelmed local systems and ensuring continued access to essential health services.^(15)^ The standardization and classification of these teams have improved the quality, coordination, and accountability of international medical deployments by ensuring that teams meet minimum operational and clinical standards. Furthermore, increasing evidence highlights the importance of multidisciplinary teamwork, structured training, and integrated response systems in improving patient outcomes and strengthening healthcare delivery during crises.^(16)^ Understanding these operational models and the evolving role of coordinated medical response teams is essential for improving disaster preparedness and strengthening the ability of healthcare systems to respond effectively to major emergencies and post-disaster health needs.^(17)^

This study has several limitations. First, the study was descriptive; therefore, the findings should be interpreted as an evaluation of operational performance and care delivery during emergencies rather than as evidence of clinical effectiveness in a causal or comparative context. Second, the broader impact on regional healthcare systems could not be directly measured; however, the mission may have helped reduce pressure on local healthcare facilities during the acute phase of the disaster. By providing decentralized access to medical care, humanitarian field teams may support continuity of services and potentially improve the responsiveness of local healthcare systems during periods of extreme demand.

Future longitudinal studies should include follow-up assessments of affected populations to evaluate long-term health outcomes, particularly regarding infectious disease sequelae, chronic disease management, and psychological recovery. Improving data collection methods in disaster settings, such as through the adoption of electronic medical record systems, may enhance real-time decision-making and strengthen epidemiological surveillance.

## CONCLUSION

Our findings emphasize the operational importance of multidisciplinary emergency medical teams in disaster-response settings. The results primarily demonstrate the team's ability to provide timely care, address a substantial proportion of urgent medical needs on-site, and support local healthcare services during a period of exceptional strain.

## Data Availability

The underlyng content is contained within the manuscript.
